# Regional communication and media analysis of aquaculture in Atlantic islands

**DOI:** 10.1007/s10499-023-01101-y

**Published:** 2023-04-14

**Authors:** Patrícia C. Machado, Bruno Pinto, Natacha Nogueira

**Affiliations:** 1 MARE - Marine and Environmental Sciences Centre, Faculty of Sciences, University of Lisbon, 1749-016 Lisbon, Portugal; 2 MARE - Marine and Environmental Sciences Centre/ARNET – Aquatic Research Network, Faculty of Sciences, University of Lisbon, 1749-016 Lisbon, Portugal; 3 Mariculture Center of Calheta, Directorate for the Sea, 9370-133 Calheta, Portugal; 4 Oceanic Observatory of Madeira, ARDITI, Madeira Tecnopolo, 9020-105 Funchal, Portugal; 5 CIIMAR - Interdisciplinary Center of Marine and Environmental Research, University of Porto, 4450-208 Matosinhos, Portugal

**Keywords:** Aquaculture, Media analysis, Communication, Newspapers, Public opinion, Trigger event

## Abstract

The way the media portrays aquaculture-related events can influence how this industry is perceived by the public and affect its success. Since media are an important source of public information, media content analysis has been carried out in several regions of the world. This study aimed to determine which aspects of aquaculture were more exposed and how they were discussed by regional media in the Madeira archipelago, an oceanic group of Portuguese islands. Analysis of aquaculture’s media coverage in the two most-read regional newspapers of Madeira was carried out over a 5-year period (2017 to 2021). For each news article, the assessment focused on the geographic scope, the main topics covered, the stakeholders with access to the debate, and the general tone of the article (risk/benefit framework). A total of 297 articles were analyzed. Results indicate the occurrence of trigger events that contributed to a shift in the amount of news published and in the way media framed aquaculture. In general, political and economic issues dominated the coverage, whereas social, environmental, scientific, and landscape matters received less media attention. The voice of the government was predominant throughout the 5 years in analysis and aquaculture was generally framed with a balanced tone, slightly more negative. Open and transparent communication between the stakeholders and the media is fundamental for the sustainable development of the aquaculture industry.

## Introduction

As most wild fisheries decline and human populations rise, aquaculture has been progressively contributing to the supply of fish worldwide, currently accounting for 56% of fish for human consumption (FAO 2022). According to the United Nations Sustainable Development Goals (SDGs), aquaculture activity can permeate several of these objectives, namely SDG2 (zero hunger), SDG12 (responsible consumption and production), and S14 (life below water). Target 14.7 specifically states that “by 2030 it is necessary to increase the economic benefits for small island developing states and countries of lower relative development from the sustainable use of marine resources, including through sustainable management of fisheries, aquaculture, and tourism” (The Global Goals 2022). Consumers’ awareness of the ethical, social, and environmental consequences of their purchases is becoming increasingly important (Del Giudice et al. 2018; Panda et al. 2020) and can affect public perception of the aquaculture industry (Schlag 2011).

As many people rely on media to obtain political, economic, environmental, and/or social information (Kapoor et al. 2017; Schulz et al. 2022), news content analysis has been a recurrently used method to explore the public representation of the aquaculture sector (Amberg and Hall 2008; Schlag 2011; Feucht and Zander 2016; Olsen and Osmundsen 2017; Weitzman and Bailey 2019). By framing events and issues in particular ways, the media can contribute to the public discussion of several themes and shape public opinion (Bonfadelli 2010; McCombs and Valenzuela 2020). The definition of framing is not consensual among researchers but, in general, it is correct to state that, at its core, framing is a communication process that involves presenting and defining an issue by a source (D’Angelo et al. 2019; Lindgren et al. 2022). Thus, the media act as a bridge for transmitting information but also stimulate the reader's critical position.

Throughout the years, several concerns have been raised in the media about the different aspects of aquaculture. One of the first studies was by Amberg and Hall (2008) which assessed how the media in the USA reacted to the publication of two scientific articles on the case of farmed salmon contamination (EWG 2003; Hites et al. 2004). The authors found that these articles “triggered” the media to investigate the issue, which led to changes in the number of news published in the U.S. press and in the way aquaculture was framed. During and shortly after these “trigger events,” there was a peak in the coverage of human health risks related to farmed salmon consumption. The potential negative consequences kept being reported by the media around the world, also drawing attention to the presence of dyes and additives in the feed and the overuse of antibiotics, growth hormones, and other harmful chemicals (such as pesticides) (Schlag 2011; Feucht and Zander 2016; Govaerts 2021).

Recent media studies about aquaculture show that the environmental aspect has gained greater prominence and is currently one of the three main topics addressed by the press (Schlag 2011; Feucht and Zander 2016; Olsen and Osmundsen 2017, Weitzman and Bailey 2019; Govaerts 2021). The most recurrent arguments warn about the risks of water pollution by antibiotics and other contaminants, the destruction of the ocean floor, accidental fish escapes, the introduction of diseases and parasites, genetic pollution, and potential changes in competition dynamics (habitat, food, or predation). Aquaculture has also been reported as an unsustainable activity because of its heavy dependence on fishing to produce fish feed (Feucht and Zander 2016; Olsen and Osmundsen 2017). On the other hand, some news emphasize potential positive impacts on wildlife conservation and the protection of endangered species through the reproduction of individuals destined for repopulation actions (Feucht and Zander 2016). Although sometimes portrayed as a complex expensive business that requires a high initial investment and specialized training of workers (Schlag 2011), the positive tone of news about aquaculture currently concerns economic issues. In general, it is presented as a good investment opportunity, a potential additional source of income, and as an industry with many different applications in science and technology (e.g., aquaponics, reproduction of threatened species, production of biofuel and other products from algae) (Feucht and Zander 2016; Olsen and Osmundsen 2017; Weitzman and Bailey 2019).

However, public perceptions are likely influenced by a wide combination of factors, such as culture, demographics, perceived risks for human health and the environment, the credibility of information sources, previous experiences with the industry, and also the personal level of knowledge and interest in the theme (Whitmarsh and Palmieri 2009; Bacher et al. 2014; Feucht and Zander 2016). Thus, media content does not directly represent people’s views on an issue but can provide an idea of what people may think about when considering it (McCombs 2011; Olsen and Osmundsen 2017; McCombs and Valenzuela 2020). By disproportionately emphasizing some potential impacts over others, the media may induce a distortion of the public’s perception regarding the probability of such impacts occurring (Amberg and Hall 2008). In turn, by the principles of democracy, the public holds the power to influence how conflicts are resolved at the political level, as well as the approval or rejection of legislation. In this way, the public’s behavior can improve or delay the development of aquaculture and influence its success in the economic market (Schlag 2011; Robinson 2016; Olsen and Osmundsen 2017). Fundamentally, for the sustainable development of aquaculture, the holistic management of this sector becomes essential.

### The context of aquaculture in Portugal

Portugal is a world-known country with a tradition of high seafood consumption in terms of quantity and diversity of products (INE 2021; Our World in Data 2021). The country has an extensive coastline and a vast exclusive economic zone (EEZ), presenting favorable conditions for the development of aquaculture. Although it has existed for a long time in the country, aquaculture had a slow development over the years (Almeida et al. 2015). The greatest progress of aquaculture in Portugal began when the country entered the European Union in 1986 (Ramalho and Dinis 2010; Rocha et al. 2022). Nevertheless, the country’s production had fluctuations over the years. In 2017, only 7.6% of national fish discharges corresponded to aquaculture production (REA 2019) and less than one percent of Europe’s total aquaculture production (FEAP 2020). Portuguese aquaculture focuses on the production of marine fish (mainly turbot, sea bream, and sea bass) and mollusk species (such as clams, oysters, and mussels), with most facilities located on the country’s mainland in coastal and transitional waters (estuaries and coastal lagoons) (INE 2021).

**Fig. 1:**
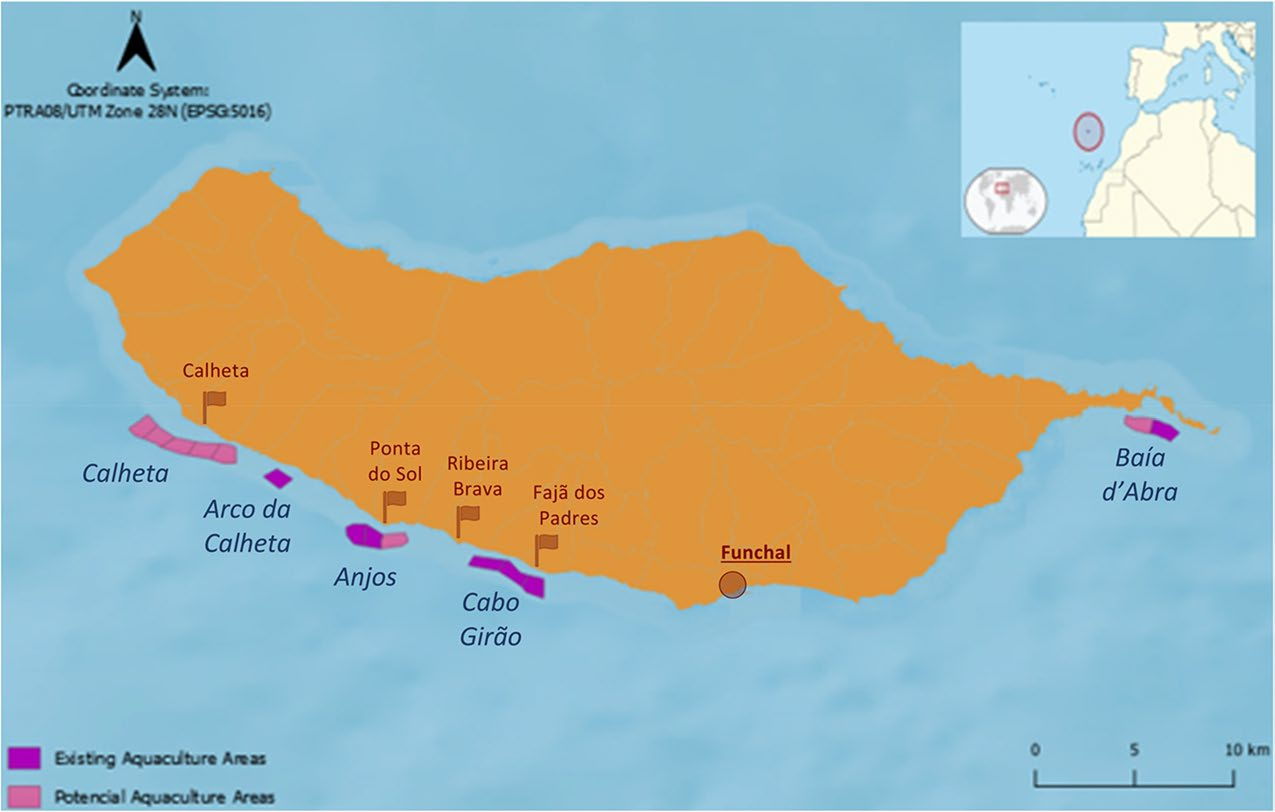
Location of Madeira Island and Zones of Interest for Aquaculture (in italics) defined in the marine spatial planning directive of 2014 (adapted from PSOEM 2019). The capital (Funchal) and the main localities involved in aquaculture (flags) are also shown

There are 1265 Portuguese licensed aquaculture establishments, mainly in the center and south of mainland Portugal, and also three based in the Madeira archipelago (INE 2021; FAO 2022). This remote group of islands located in the North Atlantic Ocean (Fig. 1) is a non-sovereign jurisdiction of the Portuguese Republic and an outermost region of the European Union. Like most islands, the Madeira archipelago is confronted with isolation and remoteness, limited natural and human resources, facing difficulties in terms of competitiveness, and economic development (United Nations 2022). They have a strong human occupation along the coastline, namely due to the existence of an extremely rugged and sloping orography, predominantly characterized by economic activities linked to the sea, such as imports and exports, fishing, and tourism. Nevertheless, the extensive maritime territory of the archipelago holds great potential for the development of marine aquaculture due to the favorable environmental conditions (e.g., water temperature), the existence of good port infrastructure, easy access by land, and good internal flow of products (PSOEM 2019). Marine aquaculture activity in Madeira started in 1996 with a pilot project of floating cages for seabream production. In recent years, efforts have been made to expand the activity. In 2016, the Regional Government defined 5 coastal areas with the greatest potential for the development of marine aquaculture, so-called ZIAs (Zones of Interest for Aquaculture), all of which are located on the south coast of Madeira Island (Fig. 1) (Resolution of the Council of Ministers no. 1025/2016; Resolution of the Council of Ministers no. 203-A/2019). To create these legal documents and guidelines, the regional government made great efforts to produce scientific knowledge, with a view to diversifying the economy, which is still heavily dependent on tourism. On the other hand, the definition of the ZIAs raised a greater demand for information on the licensing procedures and signs of political and popular contestation began to emerge. Indeed, despite the recent government initiatives to set development strategies and plans to boost the sector development, and the fact that the marine aquaculture industry is already present since the 90s, social resistance has contributed to slowing down the sector development, mostly due to conflicts between aquaculture, tourism, other sea uses, and environmental groups (Salas-Leiton et al. 2021).

In this context, the current research aimed at analyzing the regional media coverage of the aquaculture industry in the Madeira archipelago. The regional press was selected as it constitutes an integral part of local life (Quintanilha et al. 2018), generally assumed to report local events, while preserving proximity between readers and the news organization (Gonçalves et al. 2021). Newspaper selection considered the journal format, market records, and daily circulation. According to Costera Meijer (2013), regional newspapers have a local audience interested in regional news, so it may be expected that these newspapers cover more often relevant stories about their area of distribution. Research has shown that audiences of these types of newspapers are especially interested in coverage “from within,” meaning that not only close geographic events are important, but also the perspective of local people (Boukes and Vliegenthart 2017). This is even more relevant in oceanic islands, where the distinction between the wider national media (newspapers, radio, television, in traditional or digital form) and the regional press is very clear. The latter constitutes “the role of proximity irreplaceable and fundamental, an important pillar and constituent of democracy itself, which must daily reinforce and expand the space of citizenship” (Romano 2021).

Therefore, news published over a 5-year period (2017 to 2021) in two quality newspapers were examined and assessed if they present a stage for public discussion around aquaculture. This is a pioneering work in terms of aquaculture media analysis in Portugal. In particular, this study aimed to (1) identify the geographic areas mentioned by the regional newspapers, (2) recognize the main topics, (3) assess the stakeholders involved and actively contributing to the media debate around aquaculture, and finally (4) study the tones of the news concerning aquaculture.

## Methods

### News search strategy and inclusion criteria

In the Madeira archipelago, there are four regional newspapers that can all be classified as generalist quality newspapers, assuming that these types of newspapers are defined as aiming to provide comprehensive coverage and analysis of regional and national news of the day and comments on economic, political, and social issues (van Atteveldt et al. 2014). In the current market, the largest circulation is undoubtedly that of the “Diário de Notícias” (DN), followed by “Jornal da Madeira” (JN). The other two newspapers are weekly publications (“Tribuna da Madeira” and “Notícias da Madeira”), so they were excluded from the analysis as they would have less news and would present a more general view of aquaculture issues.

According to these criteria, a content analysis of news published online from 1 Jan. 2017 to 31 Dec. 2021 was carried out on the only two regional (quality) generalist daily newspapers from the Madeira archipelago: DN, a century-old newspaper that publishes about 8000 copies per day (DNotícias 2021), and JM, with an average daily circulation of about 6500 copies (JM-Madeira 2021). During this 5-year period, several events that might affect the aquaculture development strategy in Madeira occurred: the approval of the ZIAs at the end of 2016; the municipal elections in October 2017; the regional government elections in September 2019, and finally COVID-19 pandemic in March 2020. Considering that both newspapers have digital editions, data collection was done using the search engine of the newspapers’ websites with the keywords “aquaculture,” “pisciculture,” and “mariculture” (in Portuguese). All articles were then downloaded, archived, and organized in a database (Microsoft Excel 2016).

Following previous research on how the media frames aquaculture in several European countries (Feucht and Zander 2016, Olsen and Osmundsen 2017, Weitzman and Bailey 2019; Govaerts 2021), once gathered, the listed news were read thoroughly and subjected to a screening process, with the application of selection criteria defined *a priori*:

1. Aquaculture needed to be one of the two main topics of the news, i.e., there had to be a clear reference to this activity and further development of the theme;
2. Opinion articles had to express a direct or indirect critical appreciation of aquaculture;
3. If the main article was not about aquaculture, but there was a section where it was the main theme, that part of the text was included in the analysis if displaying a title of its own (for example, in the short news).

### Quantitative analysis methodology

To characterize the articles, a quantitative analysis with four different attributes was performed: (1) the *geographic scope* in which the reported events took place (where); (2) the *main topics* covered regarding aquaculture (what); (3) the *stakeholders* that participated in the debate and expressed an opinion (who); and (4) the *general tone* of the article, which placed the coverage of aquaculture in a risk/benefit framework (how) (Table 1). For these attributes, initial categories were defined based on the literature (deductive investigation methods) and additional categories were added when considered necessary (inductive investigation methods). The attribution of categories was done by two coders. In cases of doubt between categories, it was discussed among the authors until a consensus was reached (Amber and Hall 2008, Schlag 2011, Bryman 2016, Feucht and Zander 2016).

**Table 1:** Coding scheme used in the analysis of articles about aquaculture

Categories	Description
*Geographic scope*	*(Only 1 hit per article)*
International	Events take place between nations or on a European or global level;
National	Related to the total extension of the Portuguese territory;
Archipelagos	Connected to both Portuguese archipelagos (Madeira and Azores);
Madeira Regional	Linked to the total extension of the Autonomous Region of Madeira;
Local	Events refer to specific smaller areas of the territory (municipalities, civil parishes, cities, towns, villages, or other localities)
*Main topics*	*(Up to 3 hits per article)*
Economy	Monetary values, funds, and investments, financial agreements, projections of economic profits or losses, creation, or loss of jobs;
Politics	Legal documents and agreements, licenses, measures adopted, political debates, public participation, and issues related to transparency and communication;
Environment	Mentions of changes in the biophysical space (e.g., change in the quality of water or seabed) and biological communities (local fauna or flora);
Aquaculture Production	Details about the quality of aquaculture products, variety of species, production quantities, infrastructures, feed quality, diseases, and antibiotics;
Society	Social expression and manifestation, cultural values, traditions, and customs;
Landscape	Mention of esthetic and landscape impacts of aquaculture facilities;
Science and Technology	Discoveries, innovations, and information of a scientific or technological nature;
Other	The nature of the content is not included in the topics previously described and it’s scarcely covered. It includes issues about publicizing events, such as lectures, clarification sessions, forums, and congresses
*Stakeholders*	*(Unlimited number of hits per article)*
Political sphere	Party group or person with a political office who appears associated with public administration bodies;
Industry	Employee, owner, or other representatives of the industrial sector, who presents a point of view associated with the economic activity performed;
Specialist	Person or group in a scientific area of investigation or acting as an authority figure in the subject addressed (technician, expert, or academic);
Local community	Individual or association belonging to the population of the region in focus;
Other	A category that covers cases that do not fit into the main categories described;
N.A.	(Not applicable) A category used in case no actors are detected
*General tone*	*(only 1 hit per article)*
Positive	The article exclusively or mainly presents arguments that attribute value to aquaculture as an interesting activity that generates benefits;
Negative	Arguments exclusively or mostly expose aquaculture as a risky activity that entails negative impacts;
Balanced	There are both arguments for and against aquaculture, with no clear dominance between the two;
Neutral	The article is expository/informative, with no negative or positive opinions

For the *geographic scope* attribute, when in doubt, the category with the greatest spectrum was selected (e.g., between local and regional Madeira, as the latter is broader and encompasses the first, it was the category chosen). Additionally, subcategories were created within the Local category to identify specific locations. To register the *main topics*, it was defined a limit of hits *per* article, to point out the most prevalent themes. Thus, it was possible to find articles with 1, 2, or 3 main topics, resulting in a higher number of hits than articles. *Stakeholders* were defined as a person or collective who actively manifested themselves in the news, expressing an opinion or uttering words whose responsibility can be assigned to them. These words could appear as quotes or be indicated by verbs that conveyed the idea of expression. For this attribute, it was not defined a limit on the number of hits *per* article. The perception of the *general tone* of the articles was aligned with whether most of the arguments exposed in each article were for or against the development of aquaculture, in a risk/benefit framework. The classification for each article was unique so that the assigned category corresponded to the general impression conveyed by the text.

## Results

**Fig. 2:**
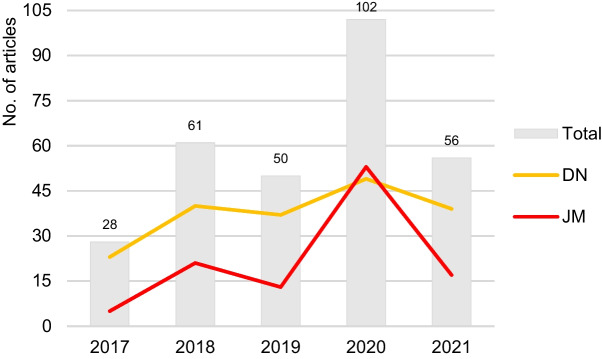
Number of news published per year and newspaper in the 5 years of analysis (*N* = 297 articles). (*Diário de Notícias* – DN and *Jornal da Madeira* – JM)

After the screening process, a total of 297 news ware selected, 188 of which belonged to the DN newspaper and 109 to the JM (Fig. 2). During the 5-year period, it was also possible to notice a peak in aquaculture coverage in 2018 and again in 2020. The latter registered the highest volume of news, with 102 articles (34% of the total sample), mostly due to an increased number of articles published by JM.

### Geographic scope

**Fig. 3:**
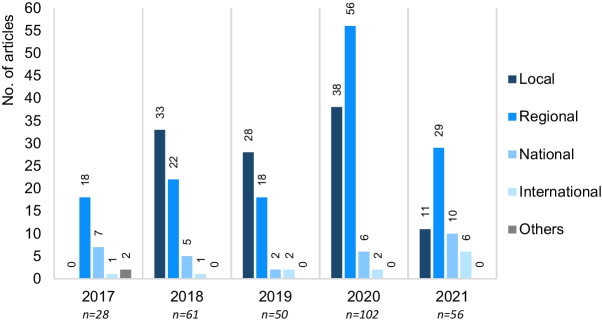
Evolution of the frequency of each category of geographic scope over the 5 years (*N* = 297 articles).The number of articles for each year is indicated on the horizontal axis

About 85% of the stories were identified as either regional (*n* = 143) or local news (*n* = 110). The remaining 15% were mostly of a national or international nature (*n* = 44) (Fig. 3). In the local scope, of the 11 municipalities of the archipelago of Madeira, the municipality of Ponta do Sol (near ZIA of Anjos) was the most conspicuous (*n* = 58), revealing its relatively important role in the discussion around the aquaculture industry in Madeira. Another three localities emerged in the analysis but with a lower frequency: Calheta, near its ZIA (*n* = 21), Ribeira Brava (*n* = 14), and Fajã dos Padres (*n* = 11) both near the ZIA of Cabo Girão, a famous touristic spot (Fig. 1). Although events reported under the regional scope were the most prevalent throughout the 5 years, in 2018 and 2019 local news coverage overlapped the volume of regional articles.

### Main topics

The analysis revealed 685 hits of main topics related to aquaculture issues. Around 70% matched four topics: politics, economy, society, and aquaculture production. In a lower ranking, topics related to environment, landscape, and science and technology represented 25% of the hits (Table 2). Some articles focused on one (*n* = 54) or two topics (*n* = 98) but most articles addressed three (*n* = 145).

**Table 2:** Number of hits *per* category of the main topic found in the articles about aquaculture. The total number of hits is highlighted in bold

Topics	Hits	
Politics	177	26%
Economy	123	18%
Society	91	13%
Aquaculture Production	87	13%
Environment	69	10%
Landscape	57	8%
Science and Technology	49	7%
Others	32	5%
Total	**685**	100%

**Fig. 4:**
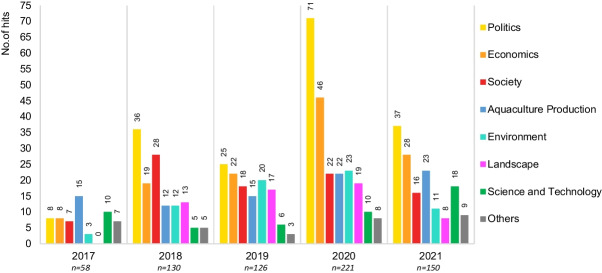
Evolution of the frequency of each main topic over the 5 years (*N* = 685 hits). The number of hits for each year is indicated on the horizontal axis

Despite the modest sample size, some heterogeneity was observed in the distribution of the topic’s frequency over time (Fig. 4). In 2017, there was not a clear prevalence of a topic over others. However, in the following year, there was a great assertion of the topic of politics, followed by Society (presenting its maximum relevance during the 5 years) and economy, together with the first appearance of the topic landscape. In 2019, the coverage of aquaculture issues became more balanced, with less contrast in the approach. 2020 registered the highest number of published articles, mostly related to political and economic issues. In 2021, the last year under analysis, the contrast between politics and economy became less evident, and articles about aquaculture production and science and technology emerged more frequently.

In general, most references consisted of political criticisms aimed at irregularities in the licensing procedure, the location and size of the floating cages, the lack of studies necessary for the correct planning and monitoring of aquaculture activity, and the existence of constraints in the compatibility of aquaculture with other economic and leisure activities. In addition, great media attention was also given to the lack of transparency in bureaucratic processes (with the consequent increase of public distrust in the licensing procedure) and the difficulty in accessing information, which obscures public involvement in political decisions.

The second most debated topic was the economy. On the one hand, aquaculture was portrayed as a profitable investment, which allows for diversification and economic growth, thus contributing to the creation of skilled jobs (not only in aquaculture but also in related industries such as manufacturing). On the other hand, it was interpreted as requiring a very large and high-risk initial investment, competing with traditional fishing and tourist activities, generating few economic advantages for the islands, and favoring only a minority.

The third most important topic was society, with a more relevant media presence from 2018 onwards. Some social benefits were recognized (e.g., contribution to the specialized technical training of the population), but aquaculture was also reported as a controversial issue, generating social discontent and a threat to the identity and customs of local communities, particularly those with tradition and dependence on fishing activities.

Aquaculture production was the fourth most frequent topic in both newspapers. This topic is characterized by information that is part of the discourse of disseminating details about the aquaculture industry. Madeira was promoted as a region with favorable conditions for aquaculture development and for the diversification of species of superior market quality. Despite some attention being drawn to possible human and animal health risks, particularly in intensive production methods, references to these subjects were scarce.

The environmental topic also became more frequent from 2018 onwards. The analyzed regional newspapers alluded to negative environmental and biodiversity impacts, such as water pollution, destruction of the seabed, genetic pollution due to accidental escapes, and the introduction of exotic species. However, it was also possible to find some references to the contribution of aquaculture to the protection of biodiversity, namely through the feeding of marine plankton with nutrients from aquaculture farms.

The Landscape was one of the least covered topics, only emerging in 2018 and increasing its relevance until 2021 when it subsided. It was often approached in conjunction with economic content, providing particular emphasis on the esthetic impact caused by the floating cages, in the Madeira maritime landscape, one of the main tourist attractions in the region. The least covered topic was science and technology which was most present only during the first and last years (2017 and 2021), highlighting the benefits of technological development in the aquaculture sector.

### Stakeholders

These agents of the debate were present in 247 (83%) articles, most of which showed only one stakeholder (*n* = 214), with some revealing two (*n* = 26), and a few with three (*n* = 5) or four (*n* = 2). In the 247 articles, a total of 289 hits were reported, with most of these belonging to the political sphere (66%) (Table 3). Stakeholders belonging to the local community, industry, and specialist categories spoke in a relatively balanced way, accounting for a total of 24% of the hits. The prevalence of the political sphere was verified continuously and uninterruptedly over the 5-year period, with the other stakeholders joining the debate from 2018 onward.

**Table 3:** Number of hits *per* category of stakeholders found in the articles about aquaculture. The total number of hits is highlighted in bold. Articles without stakeholders were excluded from this table (*n* = 50)

Stakeholders	Hits	
Political sphere	192	66%
Local community	30	10%
Other	26	9%
Industry	23	8%
Specialist	18	6%
Total	**289**	100%

### General tone

**Fig. 5:**
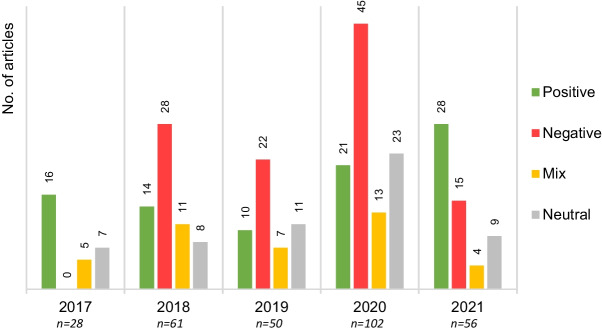
Evolution of the frequency of each general tone over the 5 years (*N* = 297 articles). The number of articles for each year is indicated on the horizontal axis

The number of articles written with a negative tone (37%) was similar to the positive ones (30%), which shows that aquaculture was framed in a relatively balanced way. Around 20% of the articles were classified as neutral and the remaining 13% had a mixed tone. Despite the modest sample size, it was noticed an emergence of a negative tone in 2018, which prevailed in the following 2 years, returning to a more positive framework in 2021 (Fig. 5).

## Discussion

This study aimed to analyze the regional media’s representation of coastal aquaculture in the Madeira archipelago and to provide insights into how the public perceives aquaculture in this region. This is the first media study about aquaculture issues in Portugal (where this industry is in expansion), which may be important to create more inclusive plans, strengthen communication among stakeholders, and improve the management of the sector. The results suggest the emergence of trigger events in 2018 and 2020 that led to an increase in the regional media attention to aquaculture.

Overall, the news mainly reported events that took place at the regional and local level, which was expected as the newspapers under analysis are written and published within the archipelago. This type of preference was also observed in the Canadian press, which recorded only 17% of national and international articles (Weitzman and Bailey 2019).

Aquaculture was approached essentially from a political and economic point of view accounting for almost half of the main topics covered. Political issues are not usually predominant in previous media studies (Schlag 2011; Feucht and Zander 2016; Govaerts 2021) and tend to be debated in a neutral and balanced way, even when they have greater media relevance (Olsen and Osmundsen 2017). However, similarly to what was found in Canada (Weitzman and Bailey 2019), Madeira regional press also gave more emphasis to the political issues, particularly on their negative aspects, addressing government transparency, the creation of legal norms around aquaculture and, a lesser extent, monitoring and enforcement. The explanation for the inconsistent importance attributed to political issues in the different media studies may be due to the existence of diverse socio-political and geographic contexts that influence how aquaculture is framed by the media (Rickard et al. 2018). According to Loja (2006), journalists from Madeira are subjected to some restrictions concerning publication freedom, due to local government action and the lack of means or will to demand the minimum conditions to fulfill their work. Local political ties with journalism tend to be powerful and compromise the quality of information, which is even more noticeable in Madeira due to its reduced territorial area (Jenkins and Nielsen 2020). The existence of bias in the treatment of information and omission of some facts may come as a result of strong political connections with local power holders and the economic interests of media owners. In fact, it is the political communication that seems to inform journalists and not journalists themselves who lead the investigation process (Neves 2022). Journalists tend to prefer sources they consider official, and these sources increasingly provide ready-to-use content (Schmitz 2011), thus acting as news-generating agents (Chaparro 2002). In the current study, the predominance of the political topic and the stakeholders from the political sphere shows how intertwined these media relations are.

In regard to the economic perspective, aquaculture was portrayed as a new economic sector with several benefits, yet great emphasis was given to the potential negative impacts that aquaculture could inflict on tourism. This is probably due to the high dependence of Madeira on the tourism sector, which accounts for 25 to 30% of the regional GDP (ARDITI 2014) and represents 78% of the regional blue economy (DREM, 2020). In previous research, the economic aspect has been exposed as the main benefit of aquaculture, generally obtaining high media attention (Höijer et al. 2006; Schlag 2011; Feucht and Zander 2016; Olsen and Osmundsen, 2017). Contrarily, Govaerts (2021) found that the French press warned about the risks of financial asphyxia and the reduced profit margins of aquaculture investment.

Following these two dominant topics, social issues and the category disclosing details about aquaculture (aquaculture production) came in third place. In other regions of the world, social issues usually do not hold as much media visibility as other topics. This may be due to the intrinsic interaction of society with politics, which explains why these topics are sometimes analyzed together (Schlag 2011; Feucht and Zander 2016). From this socio-political interdependence, issues related to conflicts of interest and communication failures are commonly highlighted. This lack of public involvement in decision-making processes noticed in Madeira is also featured in the Canadian press (Weitzman and Bailey 2019).

In contrast to the initial media studies about aquaculture, which detected a predominance of environmental and human health issues (Amberg and Hall 2008; Schlag 2011), the current research found a modest media presence of the environmental theme in Madeira. For the most part, politicians presented environmental arguments against aquaculture development and exalted the need of having updated environmental impact assessments. This means that, even when addressing environmental issues, political matters were brought into the discussion. An example of this is the conflict generated in the definition of a ZIA next to a marine protected area, in an article entitled “*Fajã dos Padres is adjacent to the newly created marine park of Cabo Girão and, as such, is not the right place for the creation of this infrastructure.*”

The landscape theme was addressed hand-in-hand with economic impacts. Issues of this nature have not yet been addressed to date in previous media studies and may be an exclusively important topic in regions where the tourism sector is of great economic importance. These matters were predominantly addressed by the political sphere but also counted with the voices of the local community. For instance, the local environmental NGO “COSMOS” expressed their concerns about the potential impact on the seascape and tourism in a newspaper article, which mentioned that “*Our beautiful maritime landscapes cannot be mischaracterized with these industrial infrastructures for fish farming, which negatively cause great landscape ‘pollution’ in our sea, which should be pristine, natural and whose horizon cannot be ‘injured and stained’ with this kind of ‘sea aviaries’.*” Furthermore, “*(aquaculture should never be) in places like Cabo Girão or other areas where the maritime landscape has to be preserved and where there are tourist facilities, because it’s these activities that represent our wealth and our livelihood.*”

As for science and technology developments, the least covered topic, despite some increased media attention in other regions of the world, results were consistent with other media studies, with this being also traditionally one of the least discussed topics (Höijer et al. 2006; Olsen and Osmundsen 2017; Weitzman and Bailey 2019).

The regional media resorted to groups of individuals who have prominent voices to enrich the news with quotes about reported events (Boyce 2006). The present results show the multiplicity of stakeholders involved in aquaculture development in Madeira and are consistent with the arguments of other authors (Feucht and Zander 2016; Osmundsen and Olsen 2017; Weitzman and Bailey 2019). Nevertheless, a category of stakeholders that is usually strongly represented is the fish farmers, which had a comparatively reduced expression in the archipelago. The controversies in Madeira were publicized as purely of a political nature, which together with the absent voice of the fish farmers, reinforced the idea that procedural and regulatory issues were at the origin of the disputes of 2018.

The articles in Madeira’s newspapers were written with a relatively balanced tone, slightly highlighting the risks of aquaculture development over its benefits. In previous media studies, the tone of articles depended on the region of the world to which they referred to. Some preferred to portray aquaculture as a benefit-generating activity (Feucht and Zander 2016; Olsen and Osmundsen 2017) while others highlighted its potential risks and losses (Weitzman and Bailey 2019; Govaerts 2021). The analysis carried out for Madeira suggests that the year 2018 marked the beginning of negative media framing, coinciding with the rise of political conflicts and social protests. This shift occurred after the municipal elections, held in October 2017, which may have contributed to triggering political conflicts and popular demonstrations in the following year. In fact, in 2018, the city council of the municipality of Ponta do Sol (next to ZIA of Anjos) denounced the expiry of the license granted for aquaculture exploration in that area. This gave rise to a chain of negative reactions to the aquaculture development in that locality and heightened regional media attention, which multiplied the number of articles published soon after and contributed to a paradigm shift in the way aquaculture was addressed. This “trigger event” phenomenon, also detected in other studies (e.g., Amberg and Hall 2008), led to an increase in articles with a Local scope. This was accompanied by a growth of the relevance of social matters, the first-time appearance of issues related to the seascape, the rise of the political and local communities’ voices, and the abrupt transition to a more negative tone in the news. Moreover, although the controversy had started in the municipality of Ponta do Sol, it spread to other municipalities with planned zones for the installation of new aquaculture cages on the seafront (ZIAs of Calheta, Arco da Calheta, and Cabo Girão). Despite the locations of the ZIAs having been already established in 2016, the regional government stressed that this plan was available for public consultation and did not receive any opinions against it at that time. Additionally, it was stated that the opinions would not be binding, as the issue of aquaculture development did not concern the municipal territory. This suggests the existence of communication failures with the public, which ultimately led to the protests of 2018. The lack of involvement of local communities in approving new projects and legal documents has been contested in the media coverage of other environmental themes, namely in the management of protected areas (Carrus et al. 2009; Castro et al. 2012). These issues have been interpreted as a violation of the principles of a democratic society and a disregard for the close relationships established between communities and the places associated with them, assuming that the reasons related to the protests are due to NIMBY arguments (“Not In My Back Yard”) (Devine-Wright 2009). These arguments are based on people-place bonds that employ a strong resistance to change, often arising when new developments disrupt pre-existing emotional attachments and threaten place-related identity processes, mostly due to its closeness (Castro et al. 2012; Devine-Wright 2009). Hence, deficient communication between the industry, the decision-makers, and the community can create conditions in which skepticism and negative attitudes toward aquaculture can turn into actions against its development (Wolsink 2007, 2012; Kraly et al. 2022).

The second “trigger event” of news about aquaculture in 2020 is probably related to the governmental political changes after the regional elections in September 2019 and the approval of the marine spatial plan by the end of 2019 (Resolution of the Council of Ministers no. 203-A/2019). Also, the world was submerged by the SARS-CoV-2 pandemic (COVID-19), which accelerated the media’s digital transformation, leading to an escalating number of digital subscriptions (and ultimately, to a higher amount of news available) (Casero-Ripollés 2021). All over the world, it became clear that readers wanted national news headlines to be put in a local context (Jolley 2020). Although this change was mostly related to subjects around the pandemic, as a side effect, journalists needed to report local (regional) matters to bridge the gap between this “news vacuum effect” and community engagement (Ardia et al. 2020). The results also suggest that, after both “trigger” events, there was a slight decrease in the volume of news published by Madeira newspapers in the years 2019 and 2021. An explanation proposed by Amberg and Hall (2008) states that the media might prefer to report recent events and reduce the publication of already known issues, a phenomenon known as “old news.”

The current study had four important limitations. One of them was the adopted timeframe of only 5 years of analysis, which may be insufficient to detect long-term trends. This resulted in a modest number of articles about aquaculture, which hindered the possibility of a more complex comparative analysis over time. A third limitation was the inexistence of previous media studies on aquaculture in Portugal, thus restricting our interpretation of results. Moreover, there is no information on public opinion on aquaculture for the Madeira archipelago or any other Portuguese region, which did not allow for a broader connection to the results obtained.

## Conclusion

In this study, aquaculture was mostly approached from a political and economic perspective, which prompted the popular and political conflicts during 2018 and 2020. Unlike other media studies on aquaculture, environmental impacts were relegated to a secondary role. Also, landscape issues were noted for the first time as a main theme in our research, which might be explained by its direct connection to tourism. However, identifying the agents that influenced the public perception of aquaculture is not an easy task and does not depend exclusively on the way the media frames themes. Indeed, it requires the ability to understand the complex factors involved in individual and collective decision-making, the formation of value judgments, and the appreciation of the context in which these decisions are made. Therefore, further analysis of Madeira’s newspapers over time is required to understand if the trends found in the current study prevailed. Questionnaires and interviews with the population can also contribute to a better understanding of the processes involved in aquaculture development. Additionally, government entities, scientists, and NGOs need to improve communication with the media, strive for a consensus and collaborate in the development of medium-term plans based on transparency and public participation. In such matters, through the endogenization and maturation of knowledge and with open and honest communication, it may be possible to contribute to the sustainable development of aquaculture, in Madeira and other regions of the world.
